# A proof-of-concept machine learning model for short-term suicide risk stratification in depressed youth

**DOI:** 10.1038/s41398-026-03944-4

**Published:** 2026-03-19

**Authors:** Bin Sun, Jie Zhang, Yarong Ma, Hongbo He

**Affiliations:** 1 Institute of Psycho-neuroscience, the Affiliated Brain Hospital, Guangzhou Medical University, Guangzhou, China; 2 Guangdong Engineering Technology Research Center for Translational Medicine of Mental Disorders, Guangzhou, China; 3 Psychosomatic Department, the Affiliated Brain Hospital, Guangzhou Medical University, Guangzhou, China; 4 Shenzhen second people’s hospital (First affiliated hospital of Shenzhen University), Shenzhen, China; 5 Guangdong Mental Health Center, Guangdong Provincial People’s Hospital, Guangdong Academy of Medical Sciences, Southern Medical University, Guangzhou, China

**Keywords:** Depression, Bipolar disorder

## Abstract

Machine learning (ML) offers promise for suicide risk stratification in depressed youth, yet its clinical application remains methodologically challenging. Using prospective data from 602 Chinese patients aged 15–24 years collected between January 2022 and June 2023, we developed ML models to predict suicide attempts within 30 days after treatment. From 102 clinical and psychosocial predictors, only 30 suicide attempts (5.0%) were observed, resulting in a limited predictor-to-event ratio. Seven algorithms were trained on 70% of the sample (*n* = 421; 21 events) using 10‑fold cross‑validation and tested on the remaining 30% (*n* = 181; 9 events), with model selection emphasizing regularization and parsimony to reduce overfitting risk. Among the algorithms, the Support Vector Machine (AUC = 0.831) and Elastic Net (AUC = 0.811) achieved the best test performance, while more complex models such as random forests and deep learning exhibited poor generalization. A combined SVM + EN ensemble reached an AUC of 0.84 in cross‑validation and identified a high‑risk decile with a 20% suicide attempt rate compared to 3.6% among remaining patients (RR = 5.53), although confidence intervals were wide due to the small number of events. These findings demonstrate the technical feasibility of ML‑based short‑term risk stratification but also underscore important methodological constraints. When retrained using only 15 LASSO-selected predictors, the model’s discrimination remained comparable (AUC = 0.82), supporting robustness against over-fitting. Low event counts limited model stability, cohort homogeneity and single‑country recruitment restricted generalizability, and the lack of temporal validation precluded assessment of model drift. Consequently, the models presented here should be viewed as proof‑of‑concept rather than evidence of clinical readiness, providing an empirical basis for future validation in larger and more diverse longitudinal cohorts.

## Introduction

Depression represents one of the leading causes of global disease burden [1], with adolescent and young adult depression posing particularly urgent public health challenges [2]. Over the past two decades, the prevalence of depression among adolescents and young adults has risen sharply, with lifetime prevalence estimates ranging from 11–25% [3, 4]. Critically, depression severity is strongly associated with suicidal behavior in this population [5, 6]. Young patients with severe or recurrent depression, especially those with prior suicide attempts or active ideation, face the highest risk [6, 7]. This risk persists acutely after clinical intervention; discharged youth with a history of suicidal behavior remain vulnerable to reattempts within one month [7, 8], underscoring the critical need for effective short-term risk stratification tools to guide post-discharge care.

Suicidal behavior arises from complex interactions among diverse factors [9], which can be broadly categorized as internalizing psychopathologies (e.g., depression, anxiety severity) [5, 7], demographic characteristics (e.g., adolescent vulnerability, gender disparities in attempt vs. completion) [10–12], externalizing psychopathologies (e.g., impulsivity, substance misuse) [13], history of suicidal/self-injurious behaviors (the strongest predictor of future attempts) [7], and social determinants (e.g., negative life events, socioeconomic stress, lack of social support) [14, 15]. This multi-factorial etiology necessitates multivariate prediction models capable of synthesizing diverse risk signals.

Machine learning (ML) has emerged as a key tool for suicide risk prediction, often leveraging large-scale electronic health records (EHRs) [8, 16, 17]. While EHR-based models demonstrate value for identifying long-term risk [8], they face limitations for immediate clinical decision-making: 1. EHRs often lack granular psychosocial, behavioral, and patient-reported outcomes crucial for short-term risk assessment ; 2. data are frequently unstructured, complicating extraction of key clinical features; 3. privacy constraints hinder data sharing and model validation. Prospective cohort studies address these gaps by systematically capturing rich, multidimensional data—including clinician assessments, validated symptom scales, personality traits, and social functioning—making them particularly suited for developing precise short-term prediction models [16].

Capitalizing on these advantages, this study utilizes data from a multi-center prospective cohort of Chinese youth with depression to:

1. Develop and validate ML models predicting suicide attempt risk within 30 days post-treatment—a critical window for intervention;
2. Integrate diverse predictors spanning sociodemographics, clinical symptoms (clinician- and self-rated), treatment adherence, self-harm history, personality traits, and family history;
3. Systematically compare algorithm performance and identify an optimized model for clinical risk stratification;
4. Categorize patients into distinct risk groups to directly inform personalized care pathways.

By bridging cohort data with advanced ML, we aim to deliver a clinically actionable tool for mitigating acute suicide risk in this vulnerable population.

## Methods

### Participants

The data for this study were obtained from a prospective multicenter cohort study, “Development and Application of a Suicide Risk Prediction Model in Adolescents with Depression”, conducted in Guangzhou, China, which originally aimed to examine the predictive effect of approximately 30 core clinical variables on baseline suicidal ideation severity (treated as a continuous variable). Participants were aged 15–24 years [18, 19] and were diagnosed with depressive episodes, recurrent depressive disorder, or bipolar disorder, current episode depressive according to ICD-10 criteria. Patients were recruited from inpatient and outpatient settings at the Affiliated Brain Hospital of Guangzhou Medical University, Guangdong Provincial People’s Hospital, and Guangdong Provincial Hospital of Traditional Chinese Medicine between January 1, 2022, and June 1, 2023. Exclusion criteria comprised: (1) inability to comprehend or complete study questionnaires; (2) physical, cognitive, or intellectual disabilities hindering participation; or (3) failure to complete the baseline assessment. Based on an empirical rule for multiple linear regression requiring 10–20 samples per predictor, and accounting for an estimated 15% attrition rate, the minimum sample size was calculated as (30 variables × 20 samples/variable) / (1 - 0.15) ≈ 753 participants. The study ultimately enrolled 777 adolescents with depression. Among 777 enrolled patients, 602 (78.1%) completed the 30-day follow-up assessing suicidal behavior and formed the final analytical sample. The remaining 175 patients were excluded as they had not yet reached the 30-day post-treatment endpoint at data cutoff. All participants provided written informed consent after receiving a detailed explanation of the study procedures. Although subsequent machine learning analyses explored an expanded set of 102 features, these analyses were conducted on a cohort whose sample size was determined a priori as sufficient for the original research objectives.

### Outcome

In this study, the outcome of interest was suicide events, including suicide attempts or suicide deaths but not suicide thoughts. After patients were enrolled and received outpatient or inpatient treatment for one month, we reached out to the patients directly or their family members, and inquired about the occurrence of the suicidal event through either a telephone interview or a WeChat video call. Respondents were asked *Since the last treatment, have you attempted suicide (i.e., deliberately harmed yourself with at least some intent to die)*? Respondents who answered affirmatively were then questioned about the frequency of such attempts and the timing of their most recent attempt. We selected a 30-day period following treatment as the timeframe for predicting suicide attempts/deaths among patients, due to the critical nature of this interval for short-term interventions and to enable comparability with prior research.

### Predictors

When selecting potential predictors, we took into account prior research on suicidal behavior and identified nine categories of factors. These factors included sociodemographic characteristics, medication treatment details, personality traits, family history of mental illness, clinical symptoms of depression and anxiety, sleep issues, symptoms of obsessive-compulsive disorder, social functioning, and thoughts and behaviors related to self-harm and suicide. The assessment tools involved include: the Clinical Global Impression Scale [20], the 17-item Hamilton Depression Rating Scale [21], the 14-item Hamilton Anxiety Rating Scale [22], the Beck Depression Inventory [23], the Beck Anxiety Inventory [24], the Yale-Brown Obsessive Compulsive Scale [25], the Adolescent Self-rating Life Events Checklist [26], the Perceived Stress Scale [27], the Athens Insomnia Scale [28], the Epworth Sleepiness Scale [29], the Hypomania Checklist-32 [30], the Ruminative Responses Scale [31], the Toronto Alexithymia Scale [32], the Self-Injurious Thoughts and Behaviors Interview [33], and the Columbia-Suicide Severity Rating Scale [34]. The predictors with missing rates exceeding 15% were excluded, while those with missing rates below 15% were imputed using k-nearest neighbor imputation. Ultimately, 102 predictors were used for the final model derivation. Among these 102 predictors, 65 are numerical variables, and 37 are categorical variables. All numerical variables were subjected to Z-score standardization prior to modeling. The detailed names of the predictive variables can be found in Table S1.

### Statistical analysis

Data were randomly split into training (70%, *n* = 421) and test (30%, *n* = 181) sets. The training set was used for model development and internal validation; the test set was reserved for external validation of the final model.

Seven machine learning algorithms were implemented and evaluated: Artificial Neural Network (ANN) [35], Support Vector Machine with radial basis function kernel (SVM) [36], Elastic Net regression (EN) [37], Naive Bayes Model (NB) [38], eXtreme Gradient Boosting (XGBoost) [39], Random Forest (RF) [40], and Deep Learning (DL) [41]. Model development and selection proceeded as follows: Optimal hyperparameters for each algorithm were determined via 10-fold cross-validation on the training set, maximizing the Area Under the Receiver Operating Characteristic Curve (AUC); the best-tuned model per algorithm was then evaluated on the independent test set using AUC. Models achieving AUC > 0.8 in both cross-validation and test evaluations were retained; ultimately, a stacking ensemble [42]. integrating these eligible models was constructed using logistic regression as the meta-learner. The ensemble’s performance was comprehensively assessed, including calculation of its AUC, sensitivity, specificity, positive predictive value (PPV), and negative predictive value (NPV) across various risk thresholds. Additionally, calibration plot and statistics (C-statistic, Eavg, Emax and Spiegelhalter’s Z-test) were used to evaluate the agreement between predicted probabilities and observed outcomes for the final model.

The top 25 predictors were reported for key individual models (e.g., EN, SVM) based on model-specific metrics (regression coefficients for EN, permutation importance for SVM).

To assess the stability of our findings following substantial predictor reduction, we conducted a supplementary analysis. Variable selection was performed exclusively within the training set to avoid information leakage. We first applied Least Absolute Shrinkage and Selection Operator (LASSO) logistic regression with ten-fold cross-validation, using the one-standard-error rule to determine the optimal regularization parameter. This procedure yielded 36 predictors with non-zero coefficients. Given that this number remained relatively large compared to our event count, we further refined the set by selecting the 15 variables with the largest absolute coefficients (based on standardized predictors). This two-step approach effectively reduced the predictor set from 102–15 while retaining the most influential variables. The resulting 15-variable set was used to retrain all seven algorithms. Each model was re-tuned using identical cross-validation procedures and subsequently evaluated on the test set (*n* = 181, 9 events) to assess performance after dimensionality reduction.

For clinical risk stratification, predicted probabilities generated by the ensemble model for the training set were ranked and divided into deciles. The observed suicide attempt rate within each decile was calculated to empirically identify thresholds separating distinct risk levels. Based on the observed distribution of event rates across deciles (e.g., identifying a point where incidence increased markedly between adjacent deciles), a threshold was operationally defined to categorize patients into a ‘High-Risk’ group (those within the highest-risk decile[s]) versus a ‘Combined Medium-Low Risk’ group (all others). This empirically derived threshold was then applied to the test set. Group differences in observed suicide attempt rates were quantified using the risk ratio (RR) with 95% confidence interval (CI) and Cohen’s d effect size with 95% CI to validate the stratification scheme.

All analyses in this study were conducted using R version 4.2.2. The packages DMwR2 and mice were used to impute missing values. The packages caret, glmnet, e1071, nnet, NeuralNetTools, and h2o were utilized for the construction of seven algorithmic models, while the caretEnsemble package was employed for model ensemble purposes. The pROC and PRROC packages were used to plot ROC curves, calculate the area under the curve (AUC), and compute metrics such as sensitivity, specificity, positive predictive value, and negative predictive value. The rms package was used to generate a calibration plot and calculate calibration statistics.

All statistical tests in this study were two-sided.

## Results

### Participant demographics

The study included 602 young patients with depression (mean age 18.9(3.2) years; 75.2% female). Within 30 days post-treatment, 30 individuals (5.0%) engaged in suicidal behavior (21 in training set, 9 in test set). Baseline characteristics were balanced between training (*n* = 421) and test (*n* = 181) sets (Table 1).

**Table 1:** Baseline characteristics of eligible visits by 602 patients to psychiatric treatment.

Characteristic		Total data	Training set	Test set
N		602	421	181
Age (mean (SD))		18.94 (3.16)	18.96 (3.23)	18.90 (3.02)
Age onset (mean (SD))		16.50 (3.34)	16.51 (3.36)	16.50 (3.28)
Patients type (%)	Inpatient	513 (85.2)	353 (83.8)	160 (88.4)
	Outpatient	89 (14.8)	68 (16.2)	21 (11.6)
Gender (%)	Male	149 (24.8)	106 (25.2)	43 (23.8)
	Female	453 (75.2)	315 (74.8)	138 (76.2)
First episode (%)		283 (47.0)	199 (47.3)	84 (46.4)
Onset form (%)	Insidious onset	579 (96.2)	404 (96.0)	175 (96.7)
	Acute onset	23 (3.8)	17 (4.0)	6 (3.3)
Education (%)	Junior high school	103 (17.1)	74 (17.6)	29 (16.0)
	Senior high school	221 (36.7)	149 (35.4)	72 (39.8)
	College or above	278 (46.2)	198 (47.0)	80 (44.2)
Relationship status (%)	Single	471 (78.2)	326 (77.4)	145 (80.1)
	In a relationship	131 (21.8)	95 (22.6)	36 (19.9)
Work (%)	Full time work	96 (15.9)	71 (16.9)	25 (13.8)
	Part time work	42 (7.0)	32 (7.6)	10 (5.5)
	Students	464 (77.1)	318 (75.5)	146 (80.7)
Payment (%)	Social medical insurance	325 (54.0)	236 (56.1)	89 (49.2)
	Self-financing	277 (46.0)	185 (43.9)	92 (50.8)
Physical disease (%)	None	411 (68.3)	283 (67.2)	128 (70.7)
	Yes (currently)	101 (16.8)	72 (17.1)	29 (16.0)
	Yes(previously)	90 (15.0)	66 (15.7)	24 (13.3)

### Model selection and ensemble

**Fig. 1:**
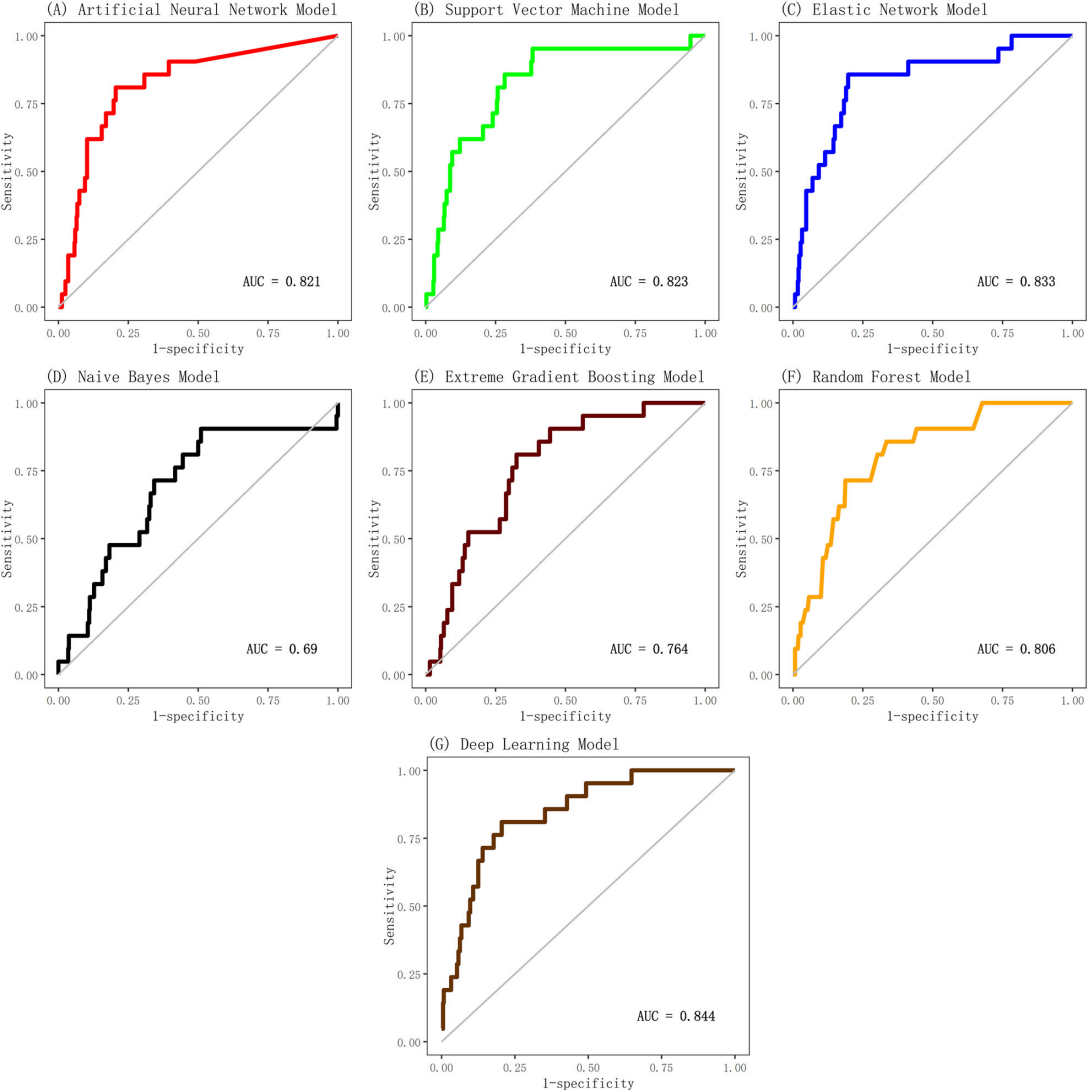
ROC curves of seven machine learning models in the cross-validation set. ROC curves illustrating the discrimination accuracy of seven machine learning algorithms for predicting suicide attempts within 30 days following psychiatric treatment in the cross-validation set, with AUC values reflecting model performance.

**Fig. 2:**
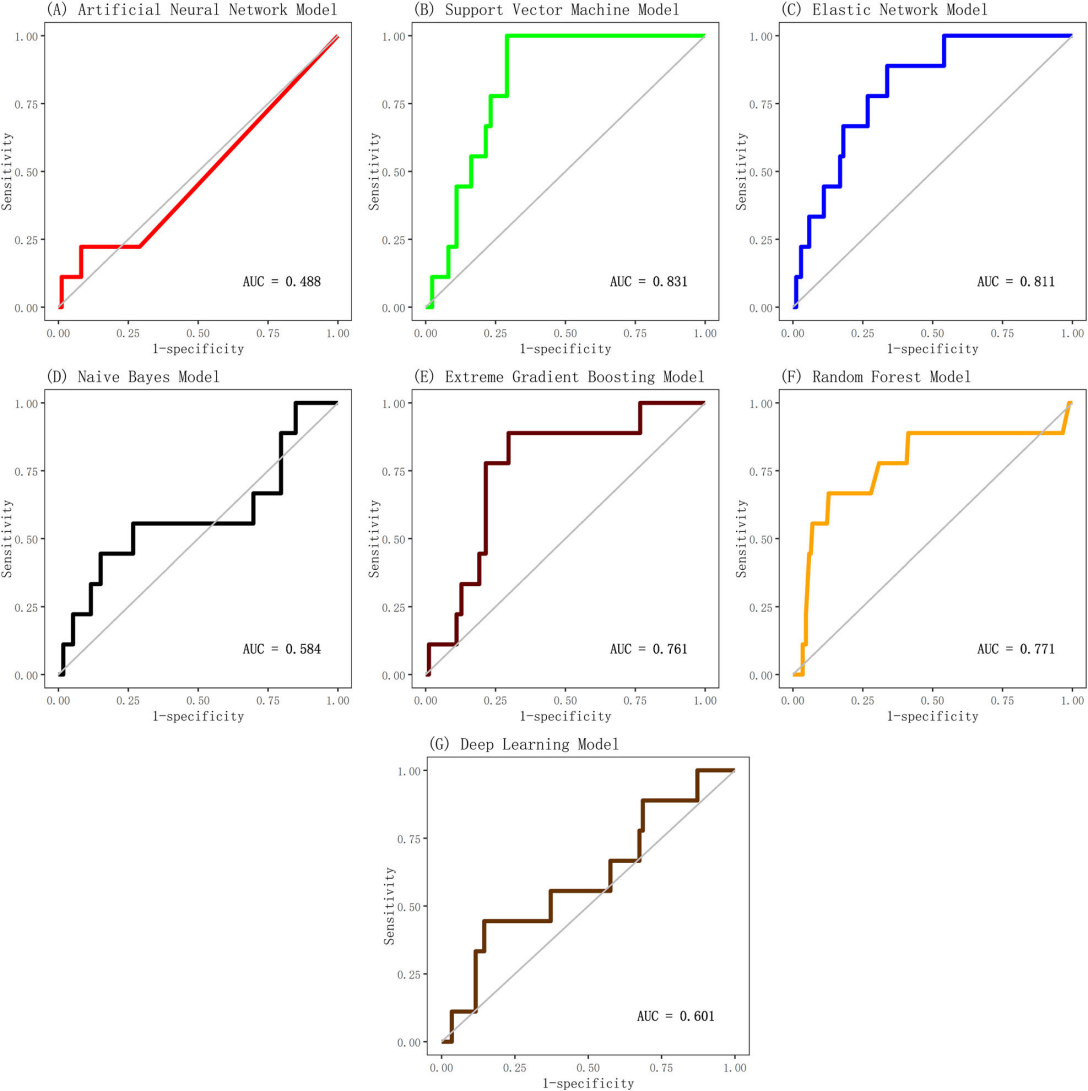
ROC curves of seven machine learning models in the independent test set. ROC curves illustrating the discrimination accuracy of seven machine learning algorithms for predicting suicide attempts within 30 days following psychiatric treatment in the independent test set, with AUC values reflecting model performance.

Among seven algorithms evaluated, only SVM (AUC_cross-validation_ = 0.823, AUC_test_ = 0.831) and EN (AUC_cross-validation_ = 0.833, AUC_test_ = 0.811) achieved AUC > 0.8 in both cross-validation (Fig. 1) and independent testing (Fig. 2). All other (ANN, RF, XGBoost, DL, NB) failed to achieve AUC > 0.8 in both cross-validation and independent testing.Stacking these two top-performing models yielded a final ensemble with a cross-validation AUC of 0.84 (Table 2).

**Table 2:** Assessment of AUCs through 10-Fold cross-validation for the optimal models Trained with support vector machine, elastic net model, and their ensemble model.

Model	ROC	ROC SD	Sensitivity (%)	Specificity (%)	Sensitivity SD (%)	Specificity SD (%)
EN	0.833	0.142	99.8	0	0.8	0
SVM	0.823	0.141	99.5	5	1.1	15.8
EN + SVM	0.840	0.119	99.3	10	1.2	21.1

*EN* elastic net model, *SVM* support vector machine.

### Model calibration

**Fig. 3:**
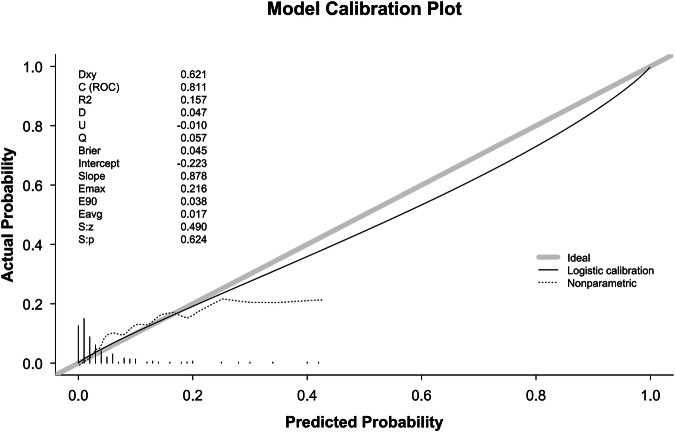
Calibration plot of the SVM + EN ensemble model. Calibration plot showing the agreement between predicted probabilities and observed suicide attempt outcomes for the support vector machine (SVM) and elastic net (EN) ensemble model, with the logistic calibration curve approximating the ideal line of perfect calibration.

The calibration plot for the prediction model is presented in Fig. 3. The model demonstrated a discriminative ability, with a C-statistic of 0.811. The logistic calibration curve closely approximated the ideal line representing perfect calibration across most of the predicted probability range. Key calibration metrics included an average absolute error (Eavg) of 0.017 and a maximum absolute error (Emax) of 0.216. The Spiegelhalter’s test for calibration yielded a non-significant p-value of 0.624. Overall, these results indicate that the model possesses good discriminative power and is well-calibrated.

### Stratification and validation of risk groups

**Fig. 4:**
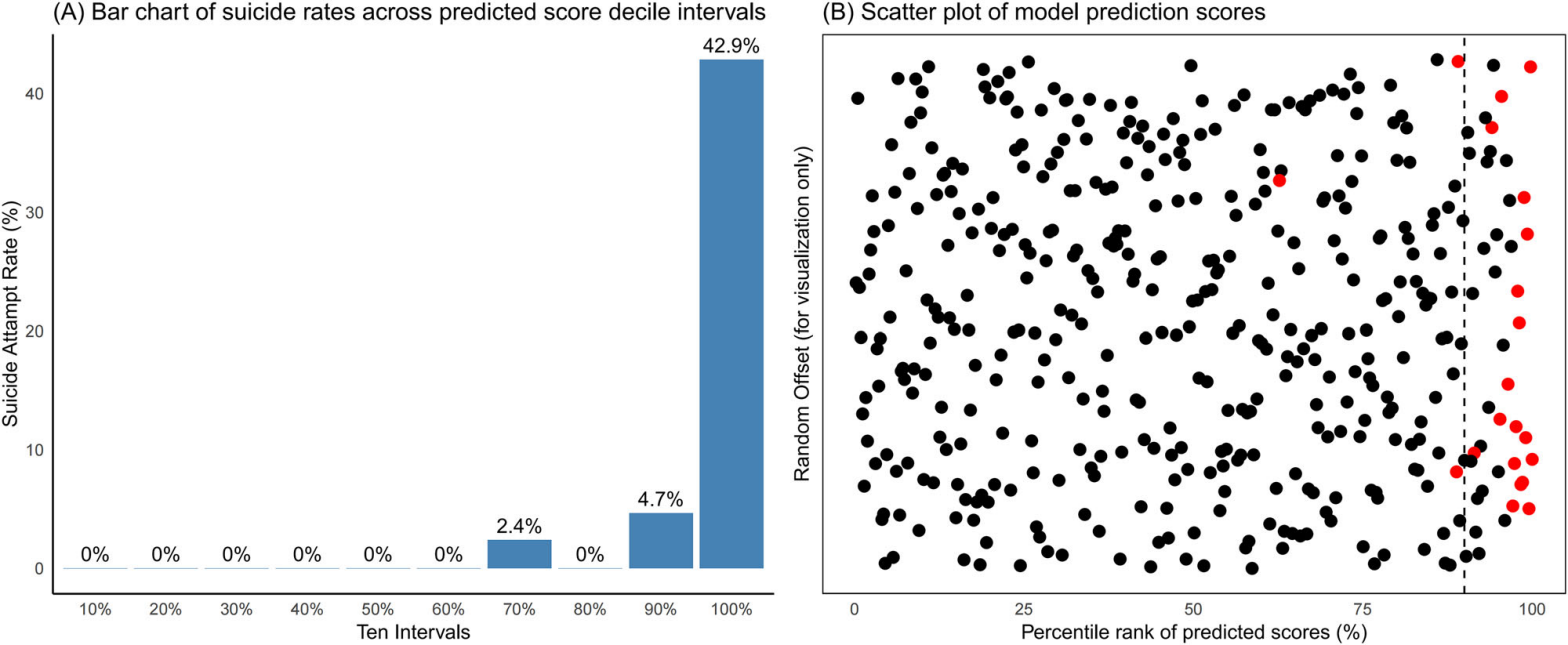
Predictive performance and score distribution of the suicide risk model. **A** Bar chart depicting the suicide attempt rate within each decile interval of the predicted risk scores. The decile intervals are displayed at the bottom of each bar. **B** Scatter plot showing the distribution of all individual predicted scores. The x-axis represents the percentile rank of these scores. A small amount of random vertical offset (jitter) has been applied to the y-axis to prevent overplotting of points; the y-axis values are arbitrary and serve visualization purposes only. The vertical dashed line marks the 90th percentile risk threshold used for risk stratification. Red dots highlight data points corresponding to individuals who experienced a suicide attempt.

Decile analysis of the ensemble’s predicted probabilities in the training set revealed a pronounced escalation in suicide attempt rates from 4.8% in the 9th decile to 42.9% in the 10th decile (Fig. 4). This distinct inflection point motivated the selection of the 90th percentile threshold (predictive score >0.121) to define a High-Risk group, while scores ≤0.121 categorized patients into a Combined Medium-Low Risk group.

**Fig. 5:**
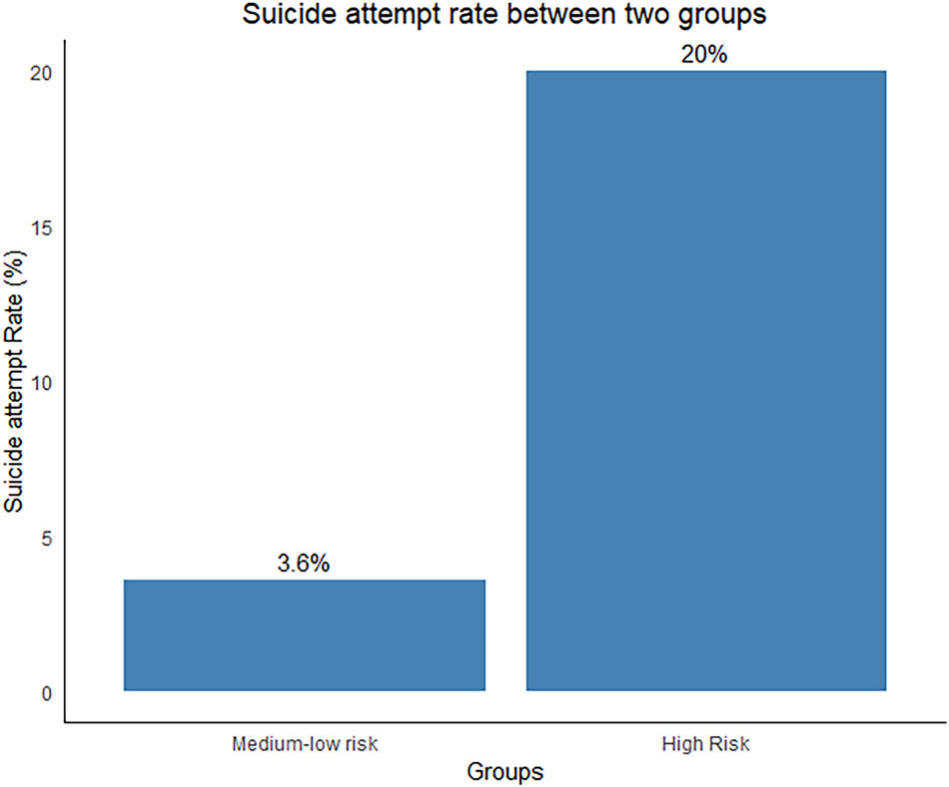
Suicide attempt rates in high-risk and medium-low risk groups. Bar chart depicting the observed suicide attempt rates in the high-risk group (90th percentile and above) and the combined medium-low risk group (below 90th percentile) in the independent test set, with the risk ratio and effect size validating the stratification utility.

When applied to the independent test set (*n* = 181), the High-Risk group exhibited a 20% suicide attempt rate, contrasting sharply with the 3.6% rate observed in the Combined Medium-Low Risk group (Fig. 5). This stratification generated a risk ratio of 5.53 (95% CI:1.384-22.125) and a Cohen’s d effect size of 0.766 (95% CI: 0.228-1.304), collectively validating the clinical utility of the model.

### Predictor importance

Table 3 lists the 25 most important predictive factors identified by the EN and SVM models, with only 10 factors overlapping between the two. The EN model prioritized behavioral determinants (e.g., alcohol use) and treatment-context variables (e.g., inpatient status), whereas the SVM emphasized historical risk factors (e.g., self-harm intent) and stress exposure (e.g., negative life events) (Table 3). Notably, depression symptom severity emerged as a consensus predictor identified by both algorithms.

**Table 3:** Predictors ranked top 25 by the best models of elastic net regression and support vector machine.

Predictors	EN	SVM
Alcohol consumption	1^st^	13^th^
Reasons for self-harm—To die	15^th^	1^st^
Onset form (acute onset)	2^nd^	
Age of starting medication		2^nd^
Patient type (inpatient)	3^rd^	
Number of hospitalizations	23^th^	3^rd^
Family history (with mental illness, but specific details unknown)	4^th^	
Reasons for self-harm—To seek attention	24^th^	4^th^
Education (college or above)	5^th^	
Other (Total score on Adolescent Life Events Scale)		5^th^
During treatment, did you decrease the dosage or stop taking medication without informing the doctor when you felt symptoms worsen or other symptoms appeared?	6^th^	
Number of manic episodes	20^th^	6^th^
Current primary diagnosis (bipolar disorder, currently in depressive episode)	7^th^	
Reasons for self-harm—For atonement		7^th^
Relationship status (In a relationship)	8^th^	
I have ever engaged in any deliberate self-harm		8^th^
Self-harm behavior (Columbia Suicide Severity Rating Scale)	14th	
Ruminative Responses (Ruminative-Responses-Scale)		9^th^
Education level (college or above)	10^th^	
Loss Factors (Total score on Adolescent Life Events Scale)		10^th^
Do you find it difficult to adhere to the treatment plan?	11^th^	
History of suicidal behavior		11^th^
Employment (part-time work)	12^th^	
Beck Depression Inventory total score	17^th^	12^th^
Family history (neurotic, stress-related, and somatoform disorders)	13^th^	
When symptoms worsen or other issues arise, I have reduced or stopped taking medication on my own.		14^th^
I sometimes forget to bring my medication when I’m away from home for extended periods.		15^th^
Taking antidepressants	16^th^	
Did you take your medication yesterday?	19^th^	16^th^
Family Intimacy and Adaptability Scale total score		17^th^
The Clinical Global Impression Scale (Improvement Index)	18^th^	18^th^
History of suicide attempts (Columbia Suicide Severity Rating Scale)	21^th^	19^th^
Education level (High school)		20^th^
Gender (Female)	9^th^	21^th^
Do you find it difficult to remember to take your medication on time and in the correct dosage?	22^th^	
Interpersonal Competence Scale total score		22^th^
Rumination symptom (Ruminative Responses Scale)		23^th^
Sometimes forget to take medication		24^th^
Epworth Sleepiness Scale total score	25^th^	
Total score on Adolescent Life Events Scale		25^th^

### Clinical decision thresholds

Table 4 details ensemble performance across risk thresholds. The AUC for the ensemble model is marginally higher than that of the optimal models derived from the two individual algorithms. At the 78.5th percentile risk threshold, the Youden’s index reached its maximum value of 1.574, with a sensitivity of 76.2%, specificity of 96.6%, positive predictive value of 34.9%, and negative predictive value of 97.9%. Table 4 additionally presents the sensitivity, specificity, and predictive values across a series of risk thresholds.

**Table 4:** Model performance metrics at various risk thresholds for predicting suicide attempt 30 days following a psychiatric treatment.

Risk threshold	Sensitivity (%)	Specificity (%)	PPV (%)	NPV (%)
99^th^	9.5	99.5	50	95.4
98^th^	14.3	98.8	37.5	95.6
97^th^	14.3	97.5	23.1	95.6
96^th^	19	96.8	23.5	95.8
95^th^	23.8	96	23.8	96
90^th^	42.9	91.8	21.4	96.8
85^th^	61.9	87.5	20.6	97.8
80^th^	71.4	82.8	17.9	98.2
78.5^th*^	76.2	81.3	17.6	98.5
75^th^	76.2	77.8	15.2	98.4
70^th^	81	72.8	13.5	98.6
65^th^	85.7	67.5	12.2	98.9
60^th^	85.7	62.3	10.7	98.8
55^th^	85.7	57	9.5	98.7
50^th^	90.5	52	9	99

^*^Threshold for maximum Youden’s index.

*NPV* negative predictive value, *PPV* positive predictive value.

Model performance metrics were based on ensemble models.

### Robustness of model performance following substantial predictor reduction

To mitigate the predictor-to-event concern, LASSO selected 15 variables covering treatment behaviour, clinical severity, historical risk and socio-demographics (Table S3); these were used to re-train all seven algorithms.

Figure S3 summarizes the test set performance of the seven models retrained with the 15 LASSO-selected predictors. The Elastic Net and Support Vector Machine models remained the top performers. The ENET model demonstrated robust stability, achieving the highest AUC of 0.815, nearly identical to its full-model performance (AUC = 0.811). In contrast, the SVM model, while still the second-best performer (AUC = 0.792), showed a more noticeable decline from its original AUC of 0.831. The Random Forest model yielded moderate performance (AUC = 0.754), and all other models (XGBoost, DL, ANN, NB) had AUCs below 0.70. The stable, superior performance of Elastic Net underscores that the core predictive signals are effectively concentrated within the 15 key variables, robustly mitigating overfitting concerns.

### Missing data pattern and sensitivity analysis

A comprehensive analysis of missing data patterns was conducted. Visual inspection using a missingness heatmap (Figure S1) indicated distinct patterns of missingness across variables. Formal statistical testing against the study outcome (suicide attempt) for all predictors with missing data showed no significant associations (all P-values > 0.05; Table S2).

To evaluate the robustness of the findings, a sensitivity analysis was performed using multiple imputation by chained equations (MICE). The entire modeling pipeline was independently repeated on five imputed datasets. The ensemble model achieved a mean test AUC of 0.825 (95% CI: 0.807–0.843) across these datasets (Table S3). The density distribution of the AUC values from each imputed dataset is shown in Figure S2. For comparison, the mean test AUCs for the Elastic Net and Support Vector Machine models were 0.801 and 0.821, respectively.

## Discussion

This study leveraged data from a multicenter cohort to train and compare seven machine learning algorithms for short-term suicide risk prediction among young patients with depression. An ensemble combining support vector machine (SVM) and elastic net (EN) achieved the most stable discrimination and stratified patients into medium–low and high-risk subgroups following treatment. The model identified a high-risk subgroup (10% of the cohort) with a 20% attempt rate versus 3.6% in the remaining cohort (RR = 5.53; 95% CI: 1.38–22.13) and the moderate-large effect size (Cohen’s d = 0.77), indicating a clinically relevant risk gradient at the population level.

Regarding model performance, complex algorithms such as deep learning and random forests failed to preserve accuracy in external validation, suggesting that simpler models may generalize better in moderate-sized clinical cohorts, a finding consistent with theoretical principles relating model complexity to the event-to-feature ratio [43]. Using stringent inclusion criteria (AUC > 0.8 in both training and testing), the combined SVM + EN ensemble demonstrated stable and replicable discrimination. Although the incremental improvement in AUC was modest (0.82/0.83 → 0.84), this modest yet consistent performance indicates enhanced reliability rather than overfitting, supporting the methodological soundness of ensemble approaches in computational psychiatry [44, 45].

A fundamental methodological concern in this study is the relatively low predictor-to-event ratio, which inherently limits model stability and increases the risk of overfitting. To address this challenge, we implemented several methodological safeguards. By systematically comparing multiple algorithms, we observed that regularized methods (Elastic Net and SVM) consistently outperformed more complex models (e.g., deep learning, random forests) during external validation. This finding empirically supports the principle that algorithmic parsimony enhances generalizability when event counts are limited, consistent with theoretical recommendations for high-dimensional data settings [43]. Further reinforcing robustness, we applied nested cross-validation with hyperparameter tuning and conducted sensitivity analyses using multiple imputation. The high agreement between the primary and sensitivity results (mean test AUC = 0.825) indicates that model performance was largely unaffected by the imputation strategy. While multiple imputation cannot eliminate the structural constraint posed by a small number of outcome events, it mitigates additional information loss due to missingness and thereby helps preserve model stability under limited effective information. Nonetheless, the small event count remains an unavoidable limitation. Thus, the convergent evidence from our validation framework, coupled with the model’s robustness to drastic predictor reduction, supports the internal reliability of the findings, though caution is warranted regarding broader clinical deployment given the cohort’s limited event count.

An evaluation of risk thresholds revealed a performance profile central to the model’s potential application: consistently high negative predictive values (NPV > 90%) co-occurred with modest positive predictive values (PPV ≈ 50%). While the threshold maximizing Youden’s index offered a balance, we prioritized the 90th percentile to define a subgroup with substantially elevated absolute risk (42.9% attempt rate in training). From a clinical perspective, the high negative predictive values (>90%) suggest the model may be useful for identifying patients at low short-term risk, potentially helping clinicians prioritize monitoring and resource allocation. However, the modest positive predictive values (maximum ~50%) indicate limited precision for individual-level predictions. This combination suggests the model may be more appropriate for ruling out near-term risk rather than triggering intensive interventions. These findings support the methodological feasibility of risk stratification but should be interpreted as proof-of-concept rather than evidence of clinical deployability.

Although our modeling pipeline initially screened 102 candidate predictors, many of these variables were derived from routinely collected clinical and self -report measures (e.g., depression severity, recent self -harm, impulsivity, medication adherence). However, collecting such a comprehensive feature set is not feasible in most clinical environments due to time constraints, limited resources, and the potential assessment burden placed on both clinicians and patients. The extensive data requirements highlight an important gap between a research -optimized, data -rich model and the streamlined assessments typically available in routine care. To enhance clinical applicability, future work should aim to identify a smaller subset of the most informative and feasible predictors through data -driven feature selection and domain expert guidance. Developing low -burden, high -yield screening models that integrate seamlessly with electronic health records or brief digital questionnaires will be essential to ensure scalability and sustainability in real -world settings.

Regarding predictor patterns, the divergent predictor hierarchies between the two top-performing algorithms (EN and SVM) reflect their complementary risk-assessment paradigms, stemming from fundamental algorithmic differences. The Elastic Net model, which estimates sparse linear effects and tends to select representative variables among correlated predictors, prioritized state-dependent markers such as alcohol use (implicated in impulsivity during depressive episodes [46]) and inpatient status (reflecting symptom acuity [47]), consistent with acute risk pathways. Conversely, the SVM, utilizing a radial basis kernel to capture nonlinear decision boundaries through distinct feature weighting mechanisms, emphasized trait-stable factors like self-harm intent (representing acquired capability for lethal behavior [48]) and negative life events (activating stress-diathesis mechanisms [49]), consistent with historical risk models. This divergence suggests that the two algorithms capture complementary risk signals. Crucially, despite their differing approaches, both converged on depression symptom severity as the central modifiable mediator, reinforcing clinical evidence that symptom remission remains the primary protective factor against near-term suicidality [50, 51].

The analysis of predictor importance revealed a clinically informative distinction between non-modifiable and modifiable risk factors. While the majority of top-ranked predictors consisted of non-modifiable variables such as gender, family history, education level, and previous self-harm or suicide attempts, which remain valuable for risk identification and stratified management [52]. the identification of modifiable factors holds greater immediate relevance for intervention. These modifiable elements, including depression severity, alcohol use, medication adherence, ruminative thinking, and family intimacy, offer concrete targets for prevention strategies. For instance, medication adherence can be improved through psychoeducation and reminder systems [53]; ruminative thinking can be addressed via cognitive behavioral therapy or mindfulness-based interventions [54, 55]; and alcohol consumption along with family intimacy can be optimized through psychoeducation and family-based therapy [56, 57]. Early interventions focusing on these modifiable factors may consequently help reduce suicide risk and improve prognosis among adolescents with depressive disorders [58].

The absence of prior suicide attempt history among the top predictors contrasts with existing literature. This may reflect several factors: (i) the short 30-day outcome window emphasizing acute over historical markers; (ii) collinearity with proximal indicators such as current self-harm intent; and (iii) regularization-induced shrinkage when historical variables offer limited marginal contribution beyond correlated predictors. Despite not ranking highly, these historical factors likely contribute to the models’ overall discrimination through their correlation with selected features.

Our model was developed and validated using data from a single continuous period (January 2022 to June 2023), which precluded formal temporal validation. While an exploratory analysis of outcome incidence across the recruitment period (Supplementary Table S4) showed no significant annual variation, minor seasonal fluctuations were observed. Therefore, we cannot determine whether the predictors’ associations with suicide risk or baseline risk levels remain stable over longer time horizons. Potential temporal drift arising from changes in clinical practice, public health measures, or societal stressors warrants prospective validation in future cohorts to ensure ongoing model calibration and applicability.

The analysis of missing data patterns provided no evidence of systematic bias, as missingness in predictors was not significantly associated with the outcome, supporting the assumption of a Missing-at-Random (MAR) mechanism. Accordingly, k-nearest-neighbor imputation was used in the primary analysis, and a sensitivity analysis using multiple imputation yielded nearly identical results (AUC = 0.831 vs. 0.825), confirming the ensemble model’s robustness to different handling strategies. Although the MAR assumption appears reasonable, the possibility of Missing-Not-at-Random (MNAR) mechanisms cannot be entirely excluded, particularly for variables such as medication adherence that may relate to unmeasured factors like disorganization.

Several limitations must be considered when interpreting our findings. First, the study’s initial predictor-to-event ratio was suboptimal, which inherently raises concerns for overfitting.

Several limitations must be considered when interpreting our findings. First, the relatively low predictor-to-event ratio inherently raises concerns for overfitting. Yet LASSO reduction to 15 variables preserved the Elastic Net AUC at 0.815, indicating that discrimination is driven by a compact, clinically coherent subset rather than by high-dimensional over-fitting. Nevertheless, the absolute number of outcome events remains limited, which is a fundamental constraint on the stability and generalizability of any predictive model derived from this cohort. Second, the generalizability of our model is constrained by the demographic and geographical homogeneity of the cohort, which was recruited exclusively from Chinese clinical centers with a high proportion (75.2%) of female participants. This limits the external validity and cross-cultural applicability of our findings, underscoring the necessity for validation in diverse populations before any broader clinical consideration. Third, although the binary indicator of antidepressant treatment was available, detailed pharmacotherapy data (e.g., specific medication class, dosage, adherence dynamics, and treatment changes) were not incorporated into the model. This omission may confound risk estimation and represents an important avenue for refinement in future studies. Fourth, the data were collected within a single, continuous 18-month period, which precludes formal assessment of the model’s temporal stability, seasonal variation, or susceptibility to drift over time. Finally, while missing data patterns were consistent with a Missing-at-Random mechanism and sensitivity analyses supported the robustness of our results, the potential for unmeasured confounding, particularly for variables such as medication adherence, cannot be fully excluded.

## Conclusions

In summary, this study demonstrates that a parsimonious ensemble of elastic net and support vector machine algorithms can achieve stable, externally validated discrimination for short-term suicide risk among young patients with depression. The results highlight that regularized and interpretable models outperform more complex approaches in moderate-sized clinical datasets and can yield clinically meaningful stratification when the predictor-to-event ratio is limited. The model’s high negative predictive values suggest potential utility for identifying patients at low immediate risk, supporting early triage and resource allocation rather than precise individual prediction. Identified modifiable predictors-particularly depression severity, alcohol use, medication adherence, and family intimacy-offer actionable targets for preventive intervention. Nevertheless, the constrained event count and cohort homogeneity limit generalizability, and the absence of formal temporal validation warrants caution before clinical implementation. Future research should expand event samples, include longitudinal replication across diverse populations, and evaluate model adaptability over time to ensure robust, sustainable applications in clinical practice.

## Supplementary information

Supplementary materials

## Data Availability

The hospital record data utilized in this study contain sensitive personal health information and, due to privacy and confidentiality restrictions, are not publicly available. De-identified data may be made available to qualified researchers upon reasonable request, subject to approval by the Institutional Review Board and the Hospital Data Access Committee. Proposals for data use and access requests should be directed to Hongbo He (hongbo_he@yeah.net) and Jie Zhang (zhangjie831014@163.com). Requests for the analysis code should be directed to Bin Sun (sunbin274@yeah.net). To gain access, requesting researchers will be required to sign a data access agreement with the study sponsor (The Affiliated Brain Hospital of Guangzhou Medical University, Guangzhou, China).
