# Supplementary material for: A proof-of-concept machine learning model for short-term suicide risk stratification in depressed youth
Source: Transl Psychiatry. 2026 Mar 19;16:187. doi: 10.1038/s41398-026-03944-4 (PMC13039823; doi:10.1038/s41398-026-03944-4)
Supplement: Supplementary file 1 — Supplementary materials [file 41398_2026_3944_MOESM1_ESM.docx]

**Supplementary materials**

Figure S1. Clustered missing data pattern heatmap.

Figure S2. Distribution of AUC values from the multiple imputation sensitivity analysis.

Figure S3 Comparison of Model Performance Using the Reduced Predictor Set (Test Set ROC).

Table S1. Descriptive statistics demonstrating the comparability of the training and test sets.​

Table S2.**The Predictors and Their Importance Rank in the Reduced Elastic Net Model.**

Table S3. Missingness mechanism analysis: P-values from chi-square tests against the outcome.​

Table S4. Performance comparison of prediction models from the multiple imputation sensitivity analysis.

Table S5. Exploratory Analysis of Outcome Incidence by Time Period.

**
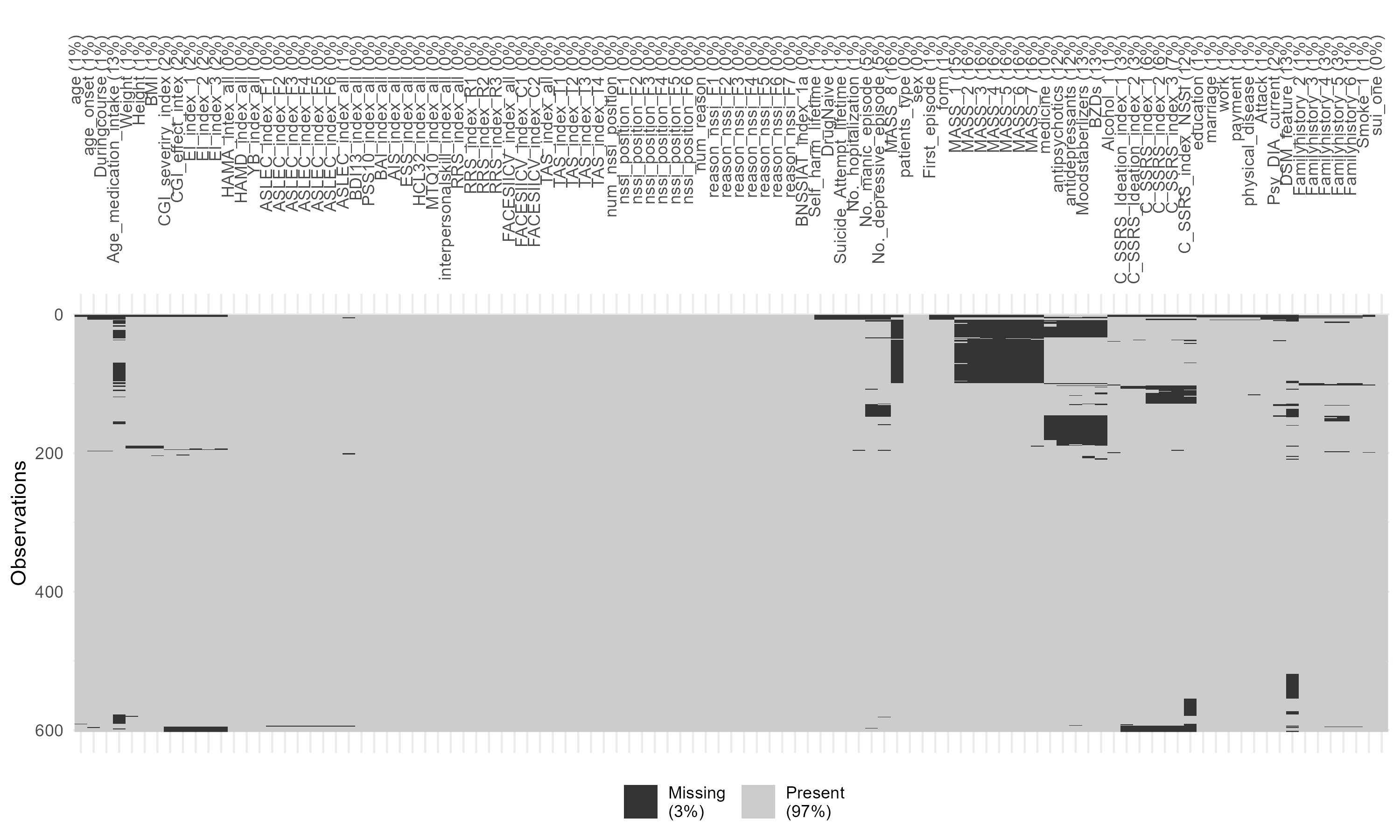
**

**Figure S1. Clustered missing data pattern heatmap**

**
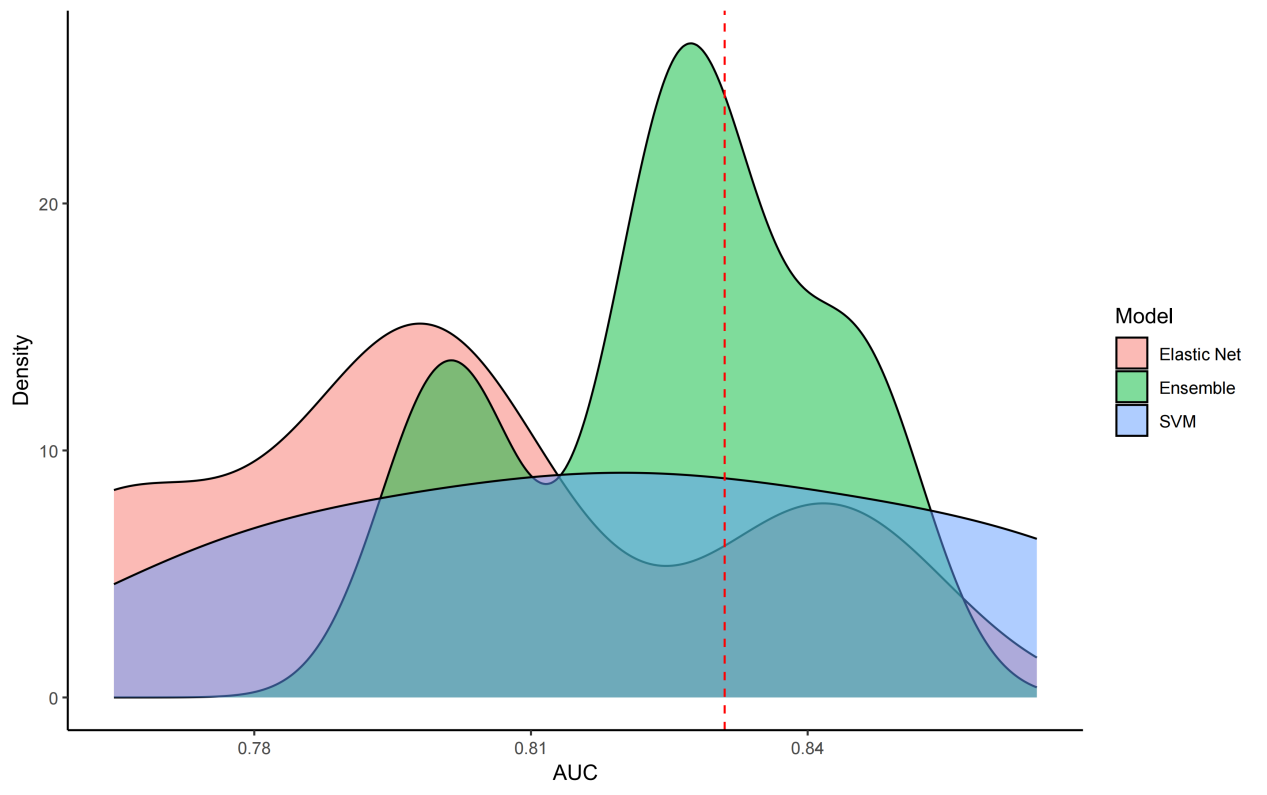
**

**Figure S2. Distribution of AUC values from the multiple imputation sensitivity analysis.** The red dashed line indicates the AUC from the primary analysis.

**
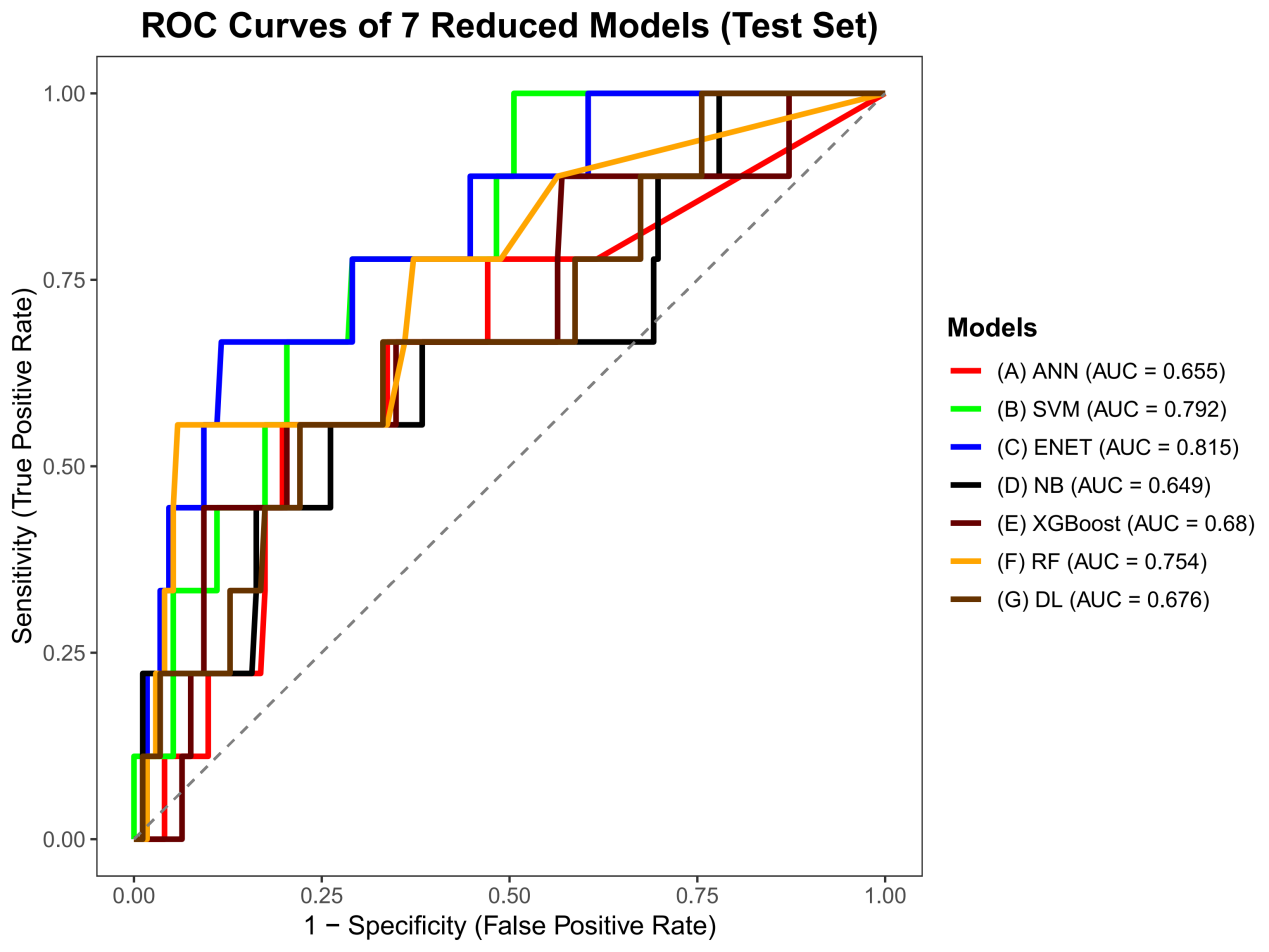
**

**Figure S3 Comparison of Model Performance Using the Reduced Predictor Set (Test Set ROC).** The seven models compared are: Artificial Neural Network (ANN), Support Vector Machine (SVM), Elastic Net (ENET), Naive Bayes (NB), Extreme Gradient Boosting (XGBoost), Random Forest (RF), and Deep Learning (DL).

**Table S1. Descriptive statistics demonstrating the comparability of the training and test sets.​**

| Characteristic | Overall | Total data | Training set | Test set |
| --- | --- | --- | --- | --- |
| n |  | 602 | 421 | 181 |
| age (mean (SD)) |  | 18.94 (3.16) | 18.96 (3.23) | 18.90 (3.02) |
| age_onset (mean (SD)) |  | 16.50 (3.34) | 16.51 (3.36) | 16.50 (3.28) |
| Duringcourse (mean (SD)) |  | 31.78 (26.93) | 31.80 (26.86) | 31.74 (27.16) |
| Age_medication_intake (mean (SD)) |  | 17.06 (3.33) | 17.03 (3.52) | 17.13 (2.84) |
| Weight (mean (SD)) |  | 56.92 (14.59) | 56.66 (14.44) | 57.50 (14.96) |
| Height (mean (SD)) |  | 163.17 (7.77) | 163.00 (7.71) | 163.56 (7.93) |
| BMI (mean (SD)) |  | 21.20 (4.78) | 21.17 (4.72) | 21.26 (4.94) |
| CGI_severiry_index (mean (SD)) |  | 4.00 (1.54) | 3.96 (1.58) | 4.07 (1.45) |
| CGI_effect_intex (mean (SD)) |  | 0.32 (0.83) | 0.32 (0.79) | 0.35 (0.92) |
| EI_index_1 (mean (SD)) |  | 1.32 (0.84) | 1.34 (0.87) | 1.27 (0.79) |
| EI_index_2 (mean (SD)) |  | 1.33 (0.85) | 1.36 (0.88) | 1.28 (0.79) |
| EI_index_3 (mean (SD)) |  | 1.03 (0.19) | 1.03 (0.20) | 1.01 (0.15) |
| HAMA_intex_all (mean (SD)) |  | 17.73 (9.67) | 17.85 (9.95) | 17.45 (8.99) |
| HAMD_index_all (mean (SD)) |  | 16.92 (8.64) | 16.96 (8.77) | 16.83 (8.34) |
| YB_index_all (mean (SD)) |  | 1.35 (4.59) | 1.29 (4.58) | 1.49 (4.61) |
| ASLEC_index_F1 (mean (SD)) |  | 11.90 (6.13) | 11.64 (6.29) | 12.50 (5.70) |
| ASLEC_index_F2 (mean (SD)) |  | 11.03 (5.79) | 10.77 (5.92) | 11.64 (5.45) |
| ASLEC_index_F3 (mean (SD)) |  | 12.11 (6.24) | 11.79 (6.39) | 12.86 (5.82) |
| ASLEC_index_F4 (mean (SD)) |  | 4.61 (2.93) | 4.57 (2.99) | 4.70 (2.79) |
| ASLEC_index_F5 (mean (SD)) |  | 8.13 (4.09) | 8.06 (4.23) | 8.29 (3.77) |
| ASLEC_index_F6 (mean (SD)) |  | 7.17 (3.92) | 6.99 (4.10) | 7.60 (3.46) |
| ASLEC_index_all (mean (SD)) |  | 53.27 (23.65) | 52.33 (24.62) | 55.45 (21.13) |
| BDI13_index_all (mean (SD)) |  | 16.26 (10.96) | 16.31 (11.11) | 16.13 (10.64) |
| PSS10_index_all (mean (SD)) |  | 17.69 (10.39) | 17.60 (10.44) | 17.91 (10.30) |
| BAI_index_all (mean (SD)) |  | 18.97 (15.27) | 19.01 (15.53) | 18.88 (14.69) |
| AIS_index_all (mean (SD)) |  | 8.68 (6.46) | 8.72 (6.52) | 8.59 (6.34) |
| ESS_index_all (mean (SD)) |  | 7.45 (6.21) | 7.44 (6.27) | 7.45 (6.10) |
| HCL32_index_all (mean (SD)) |  | 8.44 (6.85) | 7.89 (6.53) | 9.71 (7.40) |
| MTQ10_index_all (mean (SD)) |  | 23.19 (13.04) | 23.09 (13.06) | 23.44 (13.03) |
| interpersonalskill_index_all (mean (SD)) |  | 84.47 (50.78) | 84.96 (51.30) | 83.35 (49.67) |
| RRS_index_all (mean (SD)) |  | 29.16 (20.72) | 29.76 (21.11) | 27.76 (19.76) |
| RRS_index_R1 (mean (SD)) |  | 13.44 (10.68) | 13.78 (10.99) | 12.64 (9.90) |
| RRS_index_R2 (mean (SD)) |  | 10.13 (6.56) | 10.13 (6.52) | 10.15 (6.67) |
| RRS_index_R3 (mean (SD)) |  | 42.03 (30.42) | 41.48 (30.57) | 43.31 (30.14) |
| FACESIICV_index_all (mean (SD)) |  | 61.09 (36.74) | 60.47 (37.07) | 62.53 (36.03) |
| FACESIICV_index_C1 (mean (SD)) |  | 50.07 (13.48) | 50.02 (13.65) | 50.20 (13.11) |
| FACESIICV_index_C2 (mean (SD)) |  | 30.38 (14.19) | 30.22 (14.37) | 30.75 (13.78) |
| TAS_index_all (mean (SD)) |  | 59.46 (35.37) | 59.86 (35.92) | 58.53 (34.15) |
| TAS_index_T1 (mean (SD)) |  | 16.01 (10.11) | 16.05 (10.25) | 15.92 (9.81) |
| TAS_index_T2 (mean (SD)) |  | 18.35 (11.38) | 18.45 (11.55) | 18.12 (10.99) |
| TAS_index_T3 (mean (SD)) |  | 9.59 (6.39) | 9.62 (6.50) | 9.53 (6.15) |
| TAS_index_T4 (mean (SD)) |  | 15.51 (9.66) | 15.74 (9.91) | 14.97 (9.06) |
| num_nssi_position (mean (SD)) |  | 1.40 (1.84) | 1.34 (1.89) | 1.52 (1.74) |
| nssi_position_F1 (mean (SD)) |  | 0.01 (1.13) | 0.03 (1.32) | -0.05 (0.41) |
| nssi_position_F2 (mean (SD)) |  | 0.01 (1.06) | 0.00 (1.04) | 0.05 (1.09) |
| nssi_position_F3 (mean (SD)) |  | 0.03 (1.07) | 0.01 (1.06) | 0.10 (1.10) |
| nssi_position_F4 (mean (SD)) |  | 0.01 (1.01) | 0.04 (1.01) | -0.06 (0.99) |
| nssi_position_F5 (mean (SD)) |  | -0.01 (1.01) | -0.01 (1.02) | -0.03 (0.99) |
| nssi_position_F6 (mean (SD)) |  | 0.01 (1.01) | -0.04 (0.96) | 0.13 (1.12) |
| num_reason (mean (SD)) |  | 1.12 (1.52) | 1.10 (1.57) | 1.15 (1.41) |
| reason_nssi_F1 (mean (SD)) |  | 0.01 (1.02) | -0.01 (1.03) | 0.06 (1.01) |
| reason_nssi_F2 (mean (SD)) |  | 0.03 (1.13) | 0.04 (1.25) | -0.01 (0.80) |
| reason_nssi_F3 (mean (SD)) |  | 0.02 (1.04) | 0.01 (1.02) | 0.06 (1.09) |
| reason_nssi_F4 (mean (SD)) |  | 0.03 (1.10) | 0.07 (1.24) | -0.07 (0.66) |
| reason_nssi_F5 (mean (SD)) |  | 0.01 (1.11) | 0.03 (1.23) | -0.01 (0.76) |
| reason_nssi_F6 (mean (SD)) |  | -0.04 (1.05) | -0.03 (1.13) | -0.04 (0.83) |
| reason_nssi_F7 (mean (SD)) |  | 0.00 (1.04) | -0.02 (1.04) | 0.04 (1.05) |
| BNSSIAT_index_1a (mean (SD)) |  | 1.90 (1.78) | 1.90 (1.88) | 1.91 (1.52) |
| Self_harm_lifetime (mean (SD)) |  | 2.16 (0.95) | 2.14 (0.95) | 2.19 (0.94) |
| DrugNaive (mean (SD)) |  | 1.83 (0.39) | 1.83 (0.39) | 1.83 (0.37) |
| Suicide_Attempt_lifetime (mean (SD)) |  | 1.54 (0.72) | 1.55 (0.73) | 1.52 (0.70) |
| No._hopitalization (mean (SD)) |  | 0.51 (0.71) | 0.52 (0.69) | 0.50 (0.75) |
| No._manic_episode (mean (SD)) |  | 0.12 (0.38) | 0.10 (0.34) | 0.17 (0.45) |
| No._depressive_episode (mean (SD)) |  | 1.53 (0.72) | 1.54 (0.73) | 1.52 (0.68) |
| MASS_8 (mean (SD)) |  | 1.48 (0.90) | 1.46 (0.92) | 1.53 (0.86) |
| patients_type (%) | Inpatient | 513 (85.2) | 353 (83.8) | 160 (88.4) |
|  | Outpatient | 89 (14.8) | 68 (16.2) | 21 (11.6) |
| sex (%) | Male | 149 (24.8) | 106 (25.2) | 43 (23.8) |
|  | Female | 453 (75.2) | 315 (74.8) | 138 (76.2) |
| First_episode (%) | NO | 319 (53.0) | 222 (52.7) | 97 (53.6) |
|  | YES | 283 (47.0) | 199 (47.3) | 84 (46.4) |
| form (%) | Insidious onset | 579 (96.2) | 404 (96.0) | 175 (96.7) |
|  | Acute onset | 23 ( 3.8) | 17 ( 4.0) | 6 ( 3.3) |
| MASS_1 (%) | 0 | 230 (38.2) | 165 (39.2) | 65 (35.9) |
|  | 1 | 372 (61.8) | 256 (60.8) | 116 (64.1) |
| MASS_2 (%) | 0 | 304 (50.5) | 212 (50.4) | 92 (50.8) |
|  | 1 | 298 (49.5) | 209 (49.6) | 89 (49.2) |
| MASS_3 (%) | 0 | 391 (65.0) | 278 (66.0) | 113 (62.4) |
|  | 1 | 211 (35.0) | 143 (34.0) | 68 (37.6) |
| MASS_4 (%) | 0 | 442 (73.4) | 309 (73.4) | 133 (73.5) |
|  | 1 | 160 (26.6) | 112 (26.6) | 48 (26.5) |
| MASS_5 (%) | 0 | 109 (18.1) | 77 (18.3) | 32 (17.7) |
|  | 1 | 493 (81.9) | 344 (81.7) | 149 (82.3) |
| MASS_6 (%) | 0 | 402 (66.8) | 287 (68.2) | 115 (63.5) |
|  | 1 | 200 (33.2) | 134 (31.8) | 66 (36.5) |
| MASS_7 (%) | 0 | 463 (76.9) | 326 (77.4) | 137 (75.7) |
|  | 1 | 139 (23.1) | 95 (22.6) | 44 (24.3) |
| medicine (%) | 0 | 69 (11.5) | 50 (11.9) | 19 (10.5) |
|  | 1 | 533 (88.5) | 371 (88.1) | 162 (89.5) |
| antipsychotics (%) | 0 | 218 (36.2) | 148 (35.2) | 70 (38.7) |
|  | 1 | 384 (63.8) | 273 (64.8) | 111 (61.3) |
| antidepressants (%) | 0 | 148 (24.6) | 99 (23.5) | 49 (27.1) |
|  | 1 | 454 (75.4) | 322 (76.5) | 132 (72.9) |
| Moodstaberlizers (%) | 0 | 374 (62.1) | 255 (60.6) | 119 (65.7) |
|  | 1 | 228 (37.9) | 166 (39.4) | 62 (34.3) |
| BZDs (%) | 0 | 329 (54.7) | 230 (54.6) | 99 (54.7) |
|  | 1 | 273 (45.3) | 191 (45.4) | 82 (45.3) |
| Alcohol_1 (%) | 0 | 568 (94.4) | 400 (95.0) | 168 (92.8) |
|  | 1 | 34 ( 5.6) | 21 ( 5.0) | 13 ( 7.2) |
| C_SSRS_Ideation_index_1 (%) | 0 | 178 (29.6) | 127 (30.2) | 51 (28.2) |
|  | 1 | 424 (70.4) | 294 (69.8) | 130 (71.8) |
| C_SSRS_Ideation_index_2 (%) | 0 | 191 (31.7) | 136 (32.3) | 55 (30.4) |
|  | 1 | 411 (68.3) | 285 (67.7) | 126 (69.6) |
| C_SSRS_index_1 (%) | 0 | 373 (62.0) | 267 (63.4) | 106 (58.6) |
|  | 1 | 229 (38.0) | 154 (36.6) | 75 (41.4) |
| C_SSRS_index_2 (%) | 0 | 268 (44.5) | 192 (45.6) | 76 (42.0) |
|  | 1 | 334 (55.5) | 229 (54.4) | 105 (58.0) |
| C_SSRS_index_3 (%) | 0 | 366 (60.8) | 265 (62.9) | 101 (55.8) |
|  | 1 | 236 (39.2) | 156 (37.1) | 80 (44.2) |
| C_SSRS_index_NSSI (%) | 0 | 258 (42.9) | 193 (45.8) | 65 (35.9) |
|  | 1 | 344 (57.1) | 228 (54.2) | 116 (64.1) |
| education (%) | Junior high school | 103 (17.1) | 74 (17.6) | 29 (16.0) |
|  | Senior high school | 221 (36.7) | 149 (35.4) | 72 (39.8) |
|  | College | 278 (46.2) | 198 (47.0) | 80 (44.2) |
| marriage (%) | Single | 471 (78.2) | 326 (77.4) | 145 (80.1) |
|  | In love | 131 (21.8) | 95 (22.6) | 36 (19.9) |
| work (%) | Full time work | 96 (15.9) | 71 (16.9) | 25 (13.8) |
|  | Part time work | 42 ( 7.0) | 32 ( 7.6) | 10 ( 5.5) |
|  | Students | 464 (77.1) | 318 (75.5) | 146 (80.7) |
| payment (%) | Social medical insurance | 325 (54.0) | 236 (56.1) | 89 (49.2) |
|  | Self-financing | 277 (46.0) | 185 (43.9) | 92 (50.8) |
| physical_disease (%) | None | 411 (68.3) | 283 (67.2) | 128 (70.7) |
|  | Yes (currently) | 101 (16.8) | 72 (17.1) | 29 (16.0) |
|  | Yes(previously) | 90 (15.0) | 66 (15.7) | 24 (13.3) |
| Attack (%) | 1 | 517 (85.9) | 366 (86.9) | 151 (83.4) |
|  | 2 | 22 ( 3.7) | 14 ( 3.3) | 8 ( 4.4) |
|  | 3 | 63 (10.5) | 41 ( 9.7) | 22 (12.2) |
| Psy_DIA_current (%) | 1 | 534 (88.7) | 377 (89.5) | 157 (86.7) |
|  | 2 | 44 ( 7.3) | 25 ( 5.9) | 19 (10.5) |
|  | 3 | 24 ( 4.0) | 19 ( 4.5) | 5 ( 2.8) |
| DSM_feature (%) | 1 | 254 (42.2) | 175 (41.6) | 79 (43.6) |
|  | 2 | 223 (37.0) | 154 (36.6) | 69 (38.1) |
|  | 3 | 111 (18.4) | 80 (19.0) | 31 (17.1) |
|  | 4 | 14 ( 2.3) | 12 ( 2.9) | 2 ( 1.1) |
| Familyhistory_2 (%) | 1 | 597 (99.2) | 417 (99.0) | 180 (99.4) |
|  | 2 | 5 ( 0.8) | 4 ( 1.0) | 1 ( 0.6) |
| Familyhistory_3 (%) | 1 | 576 (95.7) | 403 (95.7) | 173 (95.6) |
|  | 2 | 26 ( 4.3) | 18 ( 4.3) | 8 ( 4.4) |
| Familyhistory_4 (%) | 1 | 533 (88.5) | 366 (86.9) | 167 (92.3) |
|  | 2 | 69 (11.5) | 55 (13.1) | 14 ( 7.7) |
| Familyhistory_5 (%) | 1 | 598 (99.3) | 418 (99.3) | 180 (99.4) |
|  | 2 | 4 ( 0.7) | 3 ( 0.7) | 1 ( 0.6) |
| Familyhistory_6 (%) | 1 | 570 (94.7) | 402 (95.5) | 168 (92.8) |
|  | 2 | 32 ( 5.3) | 19 ( 4.5) | 13 ( 7.2) |
| Smoke_1 (%) | 0 | 513 (85.2) | 360 (85.5) | 153 (84.5) |
|  | 1 | 18 ( 3.0) | 14 ( 3.3) | 4 ( 2.2) |
|  | 2 | 71 (11.8) | 47 (11.2) | 24 (13.3) |
| suiharm_one (%) | 0 | 461 (76.6) | 322 (76.5) | 139 (76.8) |
|  | 1 | 141 (23.4) | 99 (23.5) | 42 (23.2) |
| sui_one (%) | 0 | 572 (95.0) | 400 (95.0) | 172 (95.0) |
|  | 1 | 30 ( 5.0) | 21 ( 5.0) | 9 ( 5.0) |
| harm_one (%) | 0 | 471 (78.2) | 328 (77.9) | 143 (79.0) |
|  | 1 | 131 (21.8) | 93 (22.1) | 38 (21.0) |

**Table S2.**The Predictors and Their Importance Rank in the Reduced Elastic Net Model****

| **Predictors** | **Rank** |
| --- | --- |
| Alcohol consumption | 1 |
| During treatment, did you decrease the dosage or stop taking medication without informing the doctor when you felt symptoms worsen or other symptoms appeared? | 2 |
| Onset form (acute onset) | 3 |
| Education level (college or above) | 4 |
| Relationship status (In a relationship) | 5 |
| Gender (female) | 6 |
| Number of manic episodes | 7 |
| Patient type (inpatient) | 8 |
| Employment (part-time work) | 9 |
| Do you find it difficult to adhere to the treatment plan? | 10 |
| History of suicide attempts (Columbia Suicide Severity Rating Scale) | 11 |
| Family history (with mental illness, but specific details unknown) | 12 |
| Beck Depression Inventory total score | 13 |
| Reasons for self-harm—To die | 14 |
| Self-harm behavior (Columbia Suicide Severity Rating Scale) | 15 |

**Table S3. Missingness mechanism analysis: P-values from chi-square tests against the outcome.**

| **Variable** | **Chi_Squared** | **P_Value** | **Significance** |
| --- | --- | --- | --- |
| C_SSRS_index_2 | 1.403 | 0.236 | Not Significant |
| C_SSRS_index_3 | 1.070 | 0.301 | Not Significant |
| medicine | 0.823 | 0.364 | Not Significant |
| No._depressive_episode | 0.784 | 0.376 | Not Significant |
| No._manic_episode | 0.734 | 0.392 | Not Significant |
| Age_medication_intake | 0.673 | 0.412 | Not Significant |
| antipsychotics | 0.426 | 0.514 | Not Significant |
| C_SSRS_index_1 | 0.366 | 0.545 | Not Significant |
| Familyhistory_5 | 0.229 | 0.632 | Not Significant |
| Familyhistory_4 | 0.154 | 0.695 | Not Significant |
| BZDs | 0.087 | 0.768 | Not Significant |
| DSM_feature | 0.072 | 0.788 | Not Significant |
| Psy_DIA_current | 0.060 | 0.806 | Not Significant |
| Duringcourse | 0.027 | 0.868 | Not Significant |
| antidepressants | 0.011 | 0.915 | Not Significant |
| age_onset | 0.006 | 0.937 | Not Significant |
| MASS_1 | 0.001 | 0.973 | Not Significant |
| C_SSRS_index_NSSI | 0.000 | 0.995 | Not Significant |
| age | 0.000 | 1.000 | Not Significant |
| Weight | 0.000 | 1.000 | Not Significant |
| Height | 0.000 | 1.000 | Not Significant |
| BMI | 0.000 | 1.000 | Not Significant |
| CGI_severiry_index | 0.000 | 1.000 | Not Significant |
| CGI_effect_intex | 0.000 | 1.000 | Not Significant |
| EI_index_1 | 0.000 | 1.000 | Not Significant |
| EI_index_2 | 0.000 | 1.000 | Not Significant |
| EI_index_3 | 0.000 | 1.000 | Not Significant |
| ASLEC_index_F1 | 0.000 | 1.000 | Not Significant |
| ASLEC_index_F2 | 0.000 | 1.000 | Not Significant |
| ASLEC_index_F3 | 0.000 | 1.000 | Not Significant |
| ASLEC_index_F4 | 0.000 | 1.000 | Not Significant |
| ASLEC_index_F5 | 0.000 | 1.000 | Not Significant |
| ASLEC_index_F6 | 0.000 | 1.000 | Not Significant |
| ASLEC_index_all | 0.000 | 1.000 | Not Significant |
| Self_harm_lifetime | 0.000 | 1.000 | Not Significant |
| DrugNaive | 0.000 | 1.000 | Not Significant |
| Suicide_Attempt_lifetime | 0.000 | 1.000 | Not Significant |
| No._hopitalization | 0.000 | 1.000 | Not Significant |
| MASS_8 | 0.000 | 1.000 | Not Significant |
| First_episode | 0.000 | 1.000 | Not Significant |
| form | 0.000 | 1.000 | Not Significant |
| MASS_2 | 0.000 | 1.000 | Not Significant |
| MASS_3 | 0.000 | 1.000 | Not Significant |
| MASS_4 | 0.000 | 1.000 | Not Significant |
| MASS_5 | 0.000 | 1.000 | Not Significant |
| MASS_6 | 0.000 | 1.000 | Not Significant |
| MASS_7 | 0.000 | 1.000 | Not Significant |
| Moodstaberlizers | 0.000 | 1.000 | Not Significant |
| Alcohol_1 | 0.000 | 1.000 | Not Significant |
| C_SSRS_Ideation_index_1 | 0.000 | 1.000 | Not Significant |
| C_SSRS_Ideation_index_2 | 0.000 | 1.000 | Not Significant |
| education | 0.000 | 1.000 | Not Significant |
| marriage | 0.000 | 1.000 | Not Significant |
| work | 0.000 | 1.000 | Not Significant |
| payment | 0.000 | 1.000 | Not Significant |
| physical_disease | 0.000 | 1.000 | Not Significant |
| Attack | 0.000 | 1.000 | Not Significant |
| Familyhistory_2 | 0.000 | 1.000 | Not Significant |
| Familyhistory_3 | 0.000 | 1.000 | Not Significant |
| Familyhistory_6 | 0.000 | 1.000 | Not Significant |
| Smoke_1 | 0.000 | 1.000 | Not Significant |

**Table S4. Performance comparison of prediction models from the multiple imputation sensitivity analysis.​**

| Model | Mean_AUC | SD_AUC | CI_Lower | CI_Upper |
| --- | --- | --- | --- | --- |
| Elastic Net | 0.801 | 0.032 | 0.770 | 0.832 |
| SVM | 0.821 | 0.037 | 0.784 | 0.857 |
| Ensemble | 0.825 | 0.018 | 0.807 | 0.843 |

**Table S5. Exploratory Analysis of Outcome Incidence by Time Period.**

| Time | | Suicide Attempt (N=30) | None Suicide Attempt (N=572) | χ² | P |
| --- | --- | --- | --- | --- | --- |
| Years | 2022 | 14(5.1%) | 14(5.1%) | 0.012 | 0.911 |
|  | 2023 | 16(4.9%) | 16(4.9%) |  |  |
| Seasons | Q1 | 3(1.8%) | 161(98.2%) | 5.886 | 0.117 |
|  | Q2 | 13(7.2%) | 167(92.8%) |  |  |
|  | Q3 | 10(6.1%) | 154(93.9%) |  |  |
|  | Q4 | 4(4.3%) | 90(95.7%) |  |  |
